# The triggering mechanism for predominant hormonal signal production in fleshy fruit ripening

**DOI:** 10.1186/s43897-025-00155-1

**Published:** 2025-06-06

**Authors:** Jinyao Ouyang, Bing He, Ya Zeng, Changsheng Zhai, Yating Li, Jie Li, Pingyin Guan, Wensuo Jia

**Affiliations:** 1 College of Horticulture, China Agricultural University, Beijing, 100193 China; 2 Institute of Horticulture Crops, Xinjiang Academy of Agricultural Science, Urumqi, Xinjiang 830091 China

**Keywords:** Hormonal Signal Production, Fleshy Fruit, Ripening, Abscisic acid, Ethylene, Auxin

## Abstract

Fleshy Fruit (FF) ripening is regulated by multiple hormones, which can be categorized into two groups, i.e., the positive signals acting to promote FF ripening and the negative signals acting to suppress FF ripening. Ethylene (ET) and abscisic acid (ABA) are two predominant positive signals respectively controlling climacteric (CL) and non-climacteric (NC) FF ripening, whereas auxin (IAA) is the predominant negative signal controlling both FF growth and ripening. Functioning of these hormones is initiated by an alteration of the hormonal levels, which is referred to as the process of Hormonal Signal Production (HSP) in FF development and ripening. While the hormonal regulation of FF ripening has been extensively studied and reviewed, knowledge of HSP has never been summarized and discussed. The purpose of this review is to summarize and discuss the triggering mechanism of HSP. We first summarize the physiological, biochemical and molecular bases of HSP for three crucial hormones, ET, ABA, and IAA, including hormonal metabolism, transport and reciprocal regulation of HSP among different hormones, we then summarize and discuss the recent discoveries on the mechanism of cellular signal transduction of HSP. Finally, we propose several viewpoints to facilitate comprehension of the future research endeavors.

## Introduction

Fleshy Fruits (FF) can be categorized into two main groups, i.e., climacteric fruit (CL) that exhibits a burst of respiration and ethylene production during fruit ripening, and non-climacteric fruit (NC) that does not (Brumos 2021; Li et al. 2021; Fan et al. 2022). CL fruit ripening is controlled by ethylene (ET), whereas NC fruit ripening is controlled by ABA (Fenn and Giovannoni 2021; Li et al. 2021; Fan et al. 2022). In addition to ET and ABA, many other hormones, such as auxin (IAA), jasmonic acid (JA), brassinosteroid (BR), gibberellin (GA) and cytokinin (CTK), all play important roles in FF ripening (Fenn and Giovannoni 2021; Li et al. 2021; Perotti et al. 2023). Based on the regulatory pattern of FF ripening, these hormones can be divided into two main groups, i.e., the positive signals to promote FF ripening and the negative signals to suppress (Fenn and Giovannoni 2021; Li et al. 2021). ET, ABA and IAA have been well demonstrated to be the most important signals controlling FF ripening. Functioning of these hormones is initiated by an alteration of their levels along with FF development and ripening which is essentially a process of Hormonal Signal Production. Specifically, Hormonal Signal Production can be defined as ‘an alteration of the hormonal levels that functions to modulate fruit development and ripening’.

The physiological mechanism of HSP is determined by metabolism and transport, which are again controlled by the key enzymes or transporters (Adams-Phillips et al. 2004; Wang et al. 2022c; Jin et al. 2024), whereas the molecular mechanism of HSP is determined by a complex of signaling network, which includes primary signal perception, intermediate signaling cascades and the transcriptional or post-transcriptional regulation of the key enzymes or transporters. HSP may involve a feedback regulation, leading to hormonal signal amplification or reduction. Additionally, HSP may also involve reciprocal regulation of hormonal production, which is critical to a synergistic action of different hormones in FF ripening. Hormonal downstream signaling in FF ripening has been extensively reviewed. Here, we summarize and discuss the mechanism of HSP with an emphasis on ET, ABA and IAA, which is essentially a process of hormonal upstream signaling. We first summarize the physiological and biochemical bases of HSP, and the reciprocal regulation of HSP among differential hormones, then summarize and discuss the mechanism of signaling transduction for HSP. Finally, several new perspectives are presented to aid understanding of the prospective researches.

## Initiation of HSP determined by hormonal metabolism

**Fig. 1:**
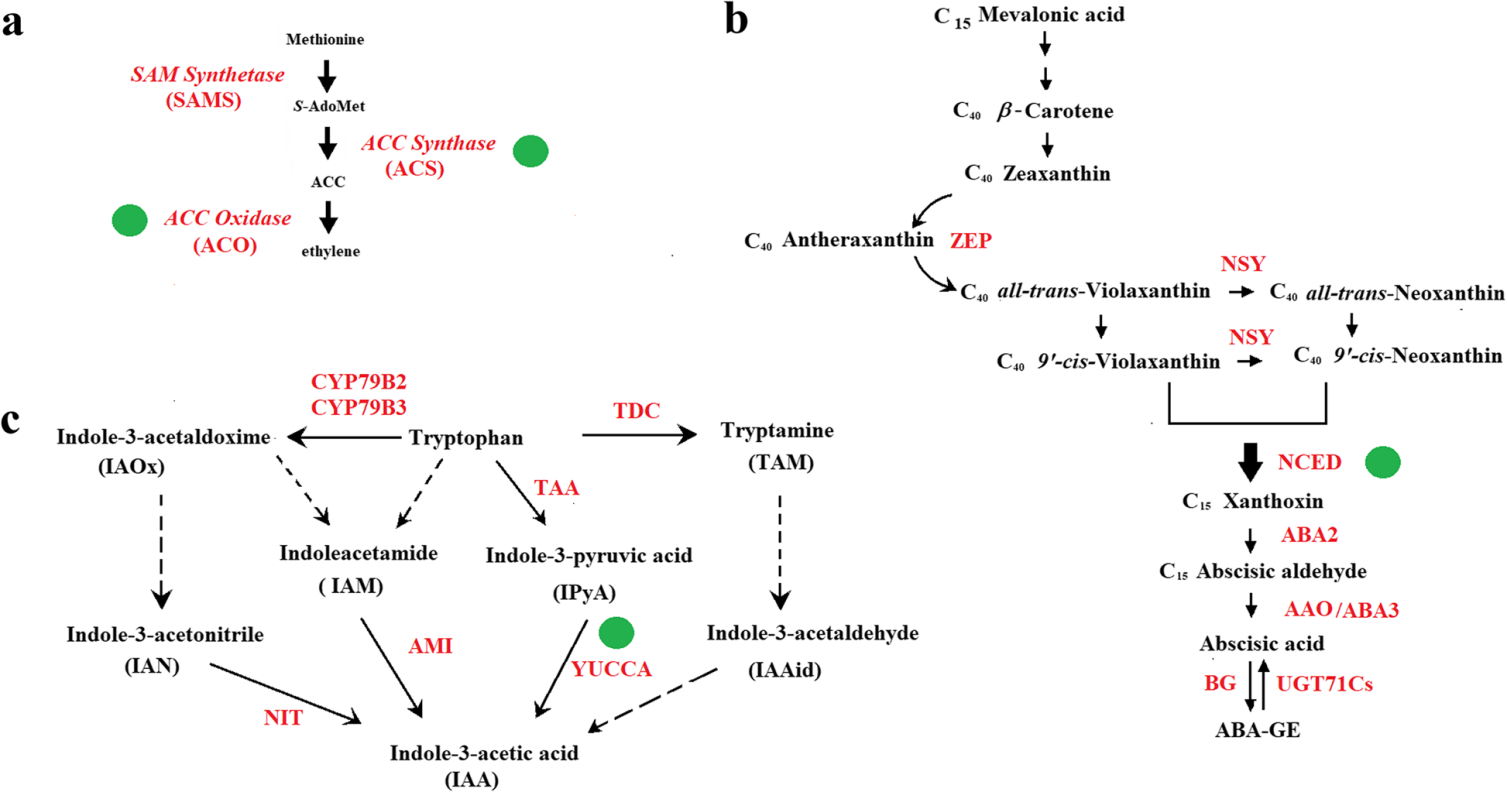
The biosynthesis pathway of hormones. The biosynthesis and catabolism pathways of hormones, showing the potential rate-limiting enzymes in each pathway. **a** ethylene biosynthesis and catabolism; **b** ABA biosynthesis and catabolism; **c** IAA biosynthesis and catabolism. The enzymes identified were shown in red. The potential rate-limiting enzymes are shown in solid green circles

HSP refers to an alteration of hormonal level, which is determined by hormonal metabolism and transport. Accordingly, identification of the key enzymes in the hormonal metabolism pathway is critical to elucidate the mechanism of HSP. Below is a summary of the current knowledge on the regulation of the key enzymes in relation to HSP, with the common knowledge on hormonal metabolic pathways simply mentioned for easier understanding of the regulatory mechanism of the key enzymes in HSP (Fig. 1).

### Ethylene

ET is derived from the amino acid methionine (Adams and Yang 1979), which is converted to S-adenosylmethionine (SAM) by SAM synthetase, then converted to 1-aminocyclopropane-1-carboxylic acid (ACC) by ACC synthases (ACS), subsequently, ACC is oxidized to ethylene by ACC oxidases (ACO) (Park et al. 2021). As thus, ACO and ACS function as two key enzymes controlling ET biosynthesis.

Accumulating studies have shown that the regulatory profile of ACS and ACO may vary among plant cultivars/varieties (Table 1). For instance, in tomato, a model of CL fruit, Chen et al. (Chen et al. 2022) investigated the expression of four members of *ACS* (i.e., *LeACS1A*, *LeACS2*, *LeACS4* and *LeACS6*) and two members of *ACO* isoforms (i.e., *LeACO2* and *LeACO3*) in two different cultivars, *S. lycopersicum* cv. Ailsa Craig and *S. pimpinellifolium*. It was shown that the transcripts of *LeACS2* and *LeACS4* substantially increased during fruit ripening in *S. pimpinellifolium*, but did not in *S. lycopersicum* cv. Ailsa Craig*.* The transcript of *LeACO3* dramatically increased at the onset of fruit ripening in both varieties, whereas *LeACO1* increased only in Ailsa Craig (Chen et al. 2022). In tomato (*Lycopersicon esculentum* Mill. cv. Momotaro), the transcript abundance of *LeACS2* and *LeACS4* was undetectable at the immature stage, but increased during the ripening stages. Conversely, *LeACS6* transcript was detectable at the immature green and mature green stages, but not in the ripening stage (Nakatsuka et al. 1998). Similarly, in the *Urebana* and *Crystal* cultivars, *LeACO1* was found to be substantially increased upon commencement of ripening, respectively, in the *Urebana* and *Crystal* cultivars (Jafari et al. 2013). Collectively, these observations appear to suggest that an increase in the *LeACS* expression is not definitely required for ET production in tomato fruit ripening. By contrast, regardless of tomato cultivars, there exists at least one member of *LeACO* that shows a dramatic increase in fruit ripening, implying the importance of *LeACO* regulation in the HSP of ET tomato fruit.

**Table 1:** Regulatory patterns of the genes in hormonal metabolism pathways during fruit ripening

Hormones	Species	Cultivars	Genes	Regulatory pattern	References
Ethylene	Tomato	*S. lycopersicum* cv. Ailsa Craig	*LeACS2/4*	Normal	(Chen et al. 2022)
			*LeACO1*	Up	
			*LeACO3*	Up	
		*S. pimpinellifolium*	*LeACS2/4*	Up	
			*LeACO1*	Normal	
			*LeACO3*	Up	
		*Solanum lycopersicum* Mill. cv. Momotaro	*LeACS2/4*	Up	(Nakatsuka et al. 1998)
			*LeACS6*	Down	
			*LeACO1/4*	Up	
		*Urebana*	*LeACO1/4*	Up	(Jafari et al. 2013)
		*Crystal*	*LeACO1/4*	Up	
	Apple	*Dongguang* × *Fuji*	*MdACS1/ACS3a*	Up	(Li et al. 2015b)
		*Jonagold*	*MdACO1*	Up	(Bulens et al. 2014)
	Pear	*Pyrus pyrifolia* Nakai. cv.Whangkeumbae	*PpACO1*	Up	(Shi and Zhang 2012)
		*Pyrus bretschneideri*	*PbACS1-8*	Up	(Cao et al. 2016)
ABA	Strawberry	*Fragaria vesca*	*FveNCED2*	Up	(Wang et al. 2017)
			*FveNCED2*	Up	(Ji et al. 2012)
			*FaNCED3/5/6*	Up	(Liao et al. 2018)
		*Fragaria chiloensis*	*FcNCED1*	Up	(Moya-León et al. 2023)
	Citrus	*Satsuma mandarin*	*CitNCED2/3*	Up	(Kato et al. 2006)
		Valencia orange (*Citrus sinensis* Osbeck)	*CitNCED3*	Up	
	Grape	*Vitis vinifera* L. cv. Muscat Hamburg	*VvNCED1*	Up	(Sun et al. 2010)
		*Vitis labrusca* L*.* × *Vitis vinifera* L. cv. Pione	*VvNCED1*	Up	(Zhang et al. 2009a)
	Bilberry	*Vaccinium myrtillus* L	*VmNCED1*	Up	(Karppinen et al. 2013)
			*VmNSY*	Up	
			*VmZEP*	Normal	
			*VmSDR/ABA2*	Normal	
	Raspberry	*Rubus idaeus* ‘Heritage’	*RiNCED1*	Up	(Álvarez et al. 2023)
	Sweet cherry	*Prunus avium* L. cv. Satonishiki	*PacNCED1*	Up	(Li et al. 2015a)
	Litchi	*Litchi chinensis* Sonn	*LcNCED1/2*	Up	(Yue et al. 2022)
	Jujube	*Z. jujuba* cultivar, ‘Dongzao’	*ZjZEP*	Up	(Zhang et al. 2019)
			*ZjNCED3*	Up	
			*ZjABA2*	Up	
			*ZjAAO3*	Up	
			*ZjABA3-1*	Up	
			*ZjABA3-2*	Up	
IAA	Strawberry	*Fragaria vesca*	*FaYUC1* (achenes)	Up	(Liu et al. 2012)
			*FaYUC1/2* (receptacle)	Down	
			*FaTAA1* (achenes)	Down	(Estrada-Johnson et al. 2017)
			*FaTAR2* (achenes)	Down	
			*FaTAR2* (achenes)	Down	
			*FaYUC2/4/7* (receptacle)	Down	
			*FaYUC1*(receptacle)	Normal	
	Peach	*Prunus persica* L. Batsch	*PpYUC6/9/11*	Down	(Pan et al. 2015)
			*PpYUC1/4/8/10*	Up	

Regulatory patterns of the genes encoding the key enzymes in hormonal metabolism in different plant species and cultivars. UP/Down/Normal denote levels of the gene expression are substantially increased, decreased or unchanged along with the progress of fruit development and ripening, respectively

In apple, a number of early studies reported that ET production was strictly related to the level of *MdACS1* expression (Sunako et al. 1999; Harada et al. 2000; Wang et al. 2009). In *Dongguang* × *Fuji*, ET production slowly increased before harvest, which was correlated to the increase of *MdACS3a* expression, whereas a dramatic increase of ET production was initiated soon after fruit harvest, which was correlated to the dramatic increase in *ACS1* expression, suggesting that different members of *ACS* were responsible for the HSP of ET at different developmental stages (Li et al. 2015b). By contrast, in ‘Jonagold’ apple, it was proposed that the burst of ET production during fruit storage was tightly coupled with the increased expression of *ACO1* (Bulens et al. 2014). Also, a recent study showed that the changing profile of the ACO activity was tightly coupled with that of ET production at the onset of fruit ripening (Giné-Bordonaba et al. 2019). In pear (*Pyrus pyrifolia* Nakai. cv. Whangkeumbae), *PpACO1* expression was limited in the immature stages, and substantially increased during fruit ripening, implying that profile of *PpACO1* was tightly linked with pear fruit ripening (Shi and Zhang 2012). In the genome of *Pyrus bretschneideri*, it was shown that none of *ACS* showed a substantial increase throughout the whole process of fruit development and ripening (Cao et al. 2016).

ACS is generally thought to be the rate-limiting enzyme in the ET biosynthesis pathway (Bulens et al. 2014; Brumos 2021; Fenn and Giovannoni 2021; Li et al. 2021; Park et al. 2021). Theoretically, the rate-limiting enzyme refers to the fact that the enzymatic activity is not capable of satisfying the need of hormonal biosynthesis under specific conditions, such that for hormonal biosynthesis to be accelerated, the enzymatic activity must be up-regulated. Therefore, to assess whether an enzyme is a rate-limiting enzyme, an important basis is to check whether the enzymatic activity would be up-regulated under the specific condition. As shown in Table 1, besides ACS, in many cases the expression of *ACO* gene is upregulated during fruit ripening. Collectively, these observations suggest that both ACS and ACO play crucial roles in the HSP of ET, whereas it can’t be concluded that it is the ACS rather than the ACO that functions to determine the HSP of ET in FF ripening.

### ABA

ABA biosynthesis proceeds with the conversion of C_40_ β-carotene into the C_40_ Zeaxanthin. As the ABA biosynthesis precursor, C_40_ β-carotene is primordially derived from the conversion of C_5_ pyruvic acid through the methylerythritol 4-phosphate (MEP) pathway. Zeaxanthin is first conversed into antheraxanthin, then converted into all-trans-violaxanthin by zeaxanthin epoxidase (ZEP). Conversion of all-trans-violaxanthin into 9-*cis*-violaxanthin or 9-*cis*-neoxanthin is believed to be a key step in ABA biosynthesis, which is catalyzed by the 9-*cis*-epoxycarotenoid dioxygenase (NCED), leading to the production of the C_15_ xanthoxin (Seo and Koshiba 2002). Finally, xanthoxin is converted to an ABA aldehyde by the short-chain alcohol dehydrogenase/reductase (SDR), and oxidation of ABA aldehyde by the ABA aldehyde oxidase (AAO) ultimately results in ABA production. Besides biosynthesis, hydrolysis of glucose-conjugated ABA (ABA-GE) to ABA by 2 β-glucosidases also contributes to elevation of ABA levels (Seo and Koshiba 2002; Dong and Hwang 2014). ABA catabolism involves two mechanisms: hydroxylation and glycosylation, which are respectively catalyzed by ABA 8’-hydroxylase (CYP707A) and abscisate β-glucosyltransferase (AOG/ABA-uridine diphosphate glucosyltransferase, ABAUGT) (Chen et al. 2020).

NCED is commonly believed to be the rate-limiting enzyme in the ABA biosynthesis pathway (Seo and Koshiba 2002; Chen et al. 2020). In diploid strawberry (*Fragaria vesca*), a model for NC fruit, it was reported that among *NCED1/2/6*, only the expression of *FveNCED*2 substantially increased during fruit ripening, was just consistent with the changing pattern of ABA levels (Ji et al. 2012; Wang et al. 2017). In contrast, in another study, the transcripts of *FaNCED3/5/6* were found to be substantially increased during fruit ripening, particularly for *FveNCED5*, which was highly expressed in receptacles, indicating that *FveNCED5* might be the predominant *NCED* gene regulating HSP of ABA (Liao et al. 2018). In *Fragaria chiloensis*, expression of *FcNCED1* and *FcNCED3* was found to be dramatically increased at the onset of fruit ripening (Moya-León et al. 2023).

Aside from strawberry, *NCED* expression was also reported to be linked with the HSP of ABA in a number of other plant cultivars/varieties. For example, in citrus, *Satsuma mandarin*, two members of *NCED*, i.e., *CitNCED2* and *CitNCED3*, were shown to be substantially increased during fruit ripening, being accompanied with a massive accumulation of ABA in the flavedo and juice sacs (Kato et al. 2006). In Valencia orange, however, while *CitNCED3* expression was increased, the level of ABA was only slightly increased in the flavedo, furthermore, neither *CitNCED2* expression nor the ABA level increased noticeably in the juice sacs. In Lisbon lemon, *CitNCED2* expression increased remarkably, corresponding with the increase of ABA levels in the flavedo and juice sacs. In grape berry (*Vitis vinifera* L. cv. Muscat Hamburg*)*, the increase in *VvNCED1* expression was reported to be tightly correlated with the increase in the ABA level during fruit ripening (Sun et al. 2010). Also, in *Vitis labrusca* L. × *Vitis vinifera* L. cv. Pione, the pattern of *VvNCED1* expression was reported to be highly consistent with the changing profile of ABA levels in fruit development and ripening (Zhang et al. 2009a). Aside from these typical NC fruits discussed above, expression of *NCED* was reported to be linked with the changes of ABA levels in fruit development and ripening in a number of other NC fruits, such as bilberry (Karppinen et al. 2013), raspberry (Álvarez et al. 2023), sweet cherry (Li et al. 2015a) and Litchi (Yue et al. 2022).

While NCED has been strongly suggested to be the key enzyme in the ABA signal production, a potential role of other enzymes in the ABA biosynthesis pathway can’t be ruled out, especially for AAO3 and ABA2, which directly catalyze the conversion of ABA from the C_15_-xanthoxin precursors downstream of NCED (Seo and Koshiba 2002; Zhang et al. 2019). In bilberry (*Vaccinium myrtillus* L.), both *VmNCED1* and *VmNSY* (neoxanthin synthase) were substantially increased at the onset of fruit ripening (Karppinen et al. 2013). In Chinese jujube, the transcripts of *ZjZEP*, *ZjNCED3*, *ZjABA2*, *ZjAAO3*, *ZjABA3-1* and *ZjABA3-2* all exhibited a dramatic increase throughout fruit ripening (Zhang et al. 2019). Ren et al. (Ren et al. 2007) elaborated the triggering mechanism for drought-induced ABA accumulation in maize, and it was demonstrated that NCED was indeed the rate-limiting enzyme at the initial stage of drought stress, but at the middle and late stage, the enzymes upstream of NCED would become the rate-limiting enzymes as a result of the depletion of the ABA precursors, ultimately determining the level of ABA accumulated. Results of this study demonstrated that the so-called ‘rate-limiting step’ is not unchanged, alternatively, it varies as the conditions (e.g. in different plant species and different developmental stages). As for the case of fruit development and ripening, this may be true in many fruit species. For example, in Chinese jujube, it was reported that the time for initial increase in the transcript of *ZjABA2*, *ZjAAO3*, *ZjABA3-1* and *ZjABA3-2* was all earlier than *ZjNCED3*, a predominant member of the NCED family.

**Fig. 2:**
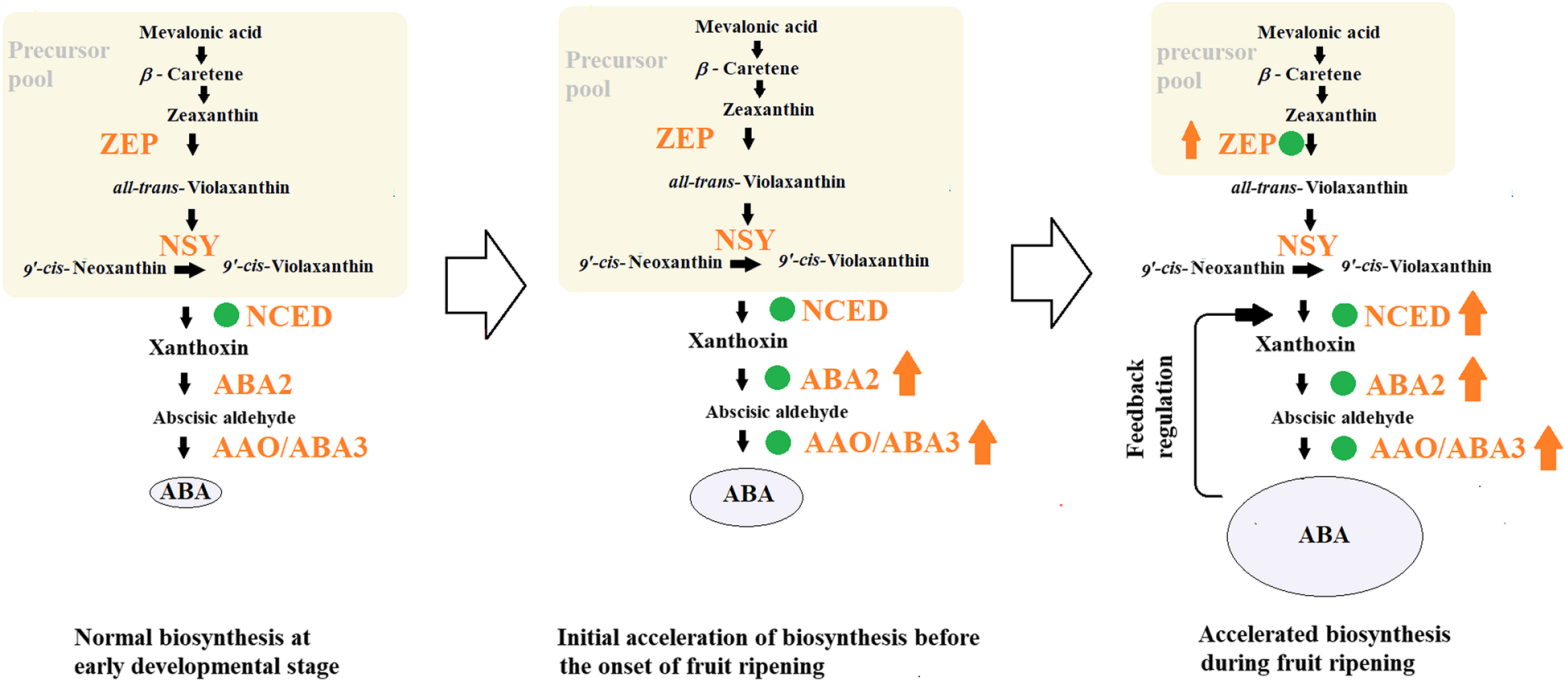
Proposed model for a regulatory pattern of the enzymes in the ABA biosynthesis pathway in fruit development and ripening. Up arrows denote up-regulation; solid green circles denote key enzymes; the size of ‘circled ABA’ denote the content of ABA in fruit cells

Here, we present a proposed model for the regulatory pattern of the enzymes in the ABA biosynthesis pathway in fruit development and ripening (Fig. 2). In the early development stage, ABA was produced at a relatively low speed because NCED is a rate-limiting enzyme, which acts to constrain the flow of the precursors of ABA biosynthesis to ABA. At a specific stage (possibly just before the onset of fruit ripening), AAO/ABA3 and ABA2, two end enzymes that function to convert xanthoxin to ABA, would be up-regulated, leading to an increase in the ABA content. The initially accumulated ABA may be able to induce NCED expression via a feedback regulation mechanism (see "Feedback regulation of hormonal biosynthesis in relation to hormonal signal amplification" section). Upregulation of the NCED activity would result in a consumption of the precursor pool, and this would ultimately require a synchronized upregulation of the enzyme upstream of NCED to satisfy the need for continuous and massive ABA production during fruit ripening.

At the early developmental stage when NCED acts to be the rate-limiting enzymes and there exists a large precursor pool upstream of NCED, ABA is synthesized at a normal. At the stage around the onset of fruit ripening, the end enzyme of the pathway, ABA3 and ABA2, may start to increase in their activity, leading to an initial ABA accumulation. The initially accumulated ABA may act to activate NCED via a positive feedback regulation mechanism, leading to accelerated consumption of the precursor pool of ABA biosynthesis. Consumption of the precursor pool would induce an up-regulation of the enzymes upstream of NCED, thus leading to a synchronized regulation of many enzymes in the pathway to satisfy the need of a continuous and massive ABA production during fruit ripening.

ABA levels are determined by a dynamic equilibration between biosynthesis and catabolism. Accordingly, to understand the mechanism for ABA signal production, a potential role of the ABA catabolism-associated enzymes must be considered. There have been reports that the catabolic rate of ABA is proportional to the level of ABA, i.e., the higher the ABA content is, the quicker the ABA catabolism is, such that the ABA catabolism will contribute to avoiding detrimental effects on plant growth and development due to over-accumulation of ABA (Griesser et al. 2020). In grape berries, the content of ABA-GE was strongly increased at veraison and peaked during post-veraison (Griesser et al. 2020). Further, the expression of *VviUGT73B4*, which catalyzes the conversion of ABA to ABA-GE, was strongly increased at the veraison. As the formation of ABA-GE will contribute to a decrease of ABA accumulation, regulation BG is logically not involved in the ABA signal production. In strawberry, reports on the regulation of the ABA 8’-hydroxylase (CYP707A) pathway showed controversial results. For example, Griesser et al. (Griesser et al. 2020) reported that the content of phaseic acid (PA), and dihydrophaseic acid (DPA) was decreased at the veraison stage. By contrast, Figueroa et al. (Figueroa et al. 2021) reported that dihydrophaseic acid (DPA) content increased throughout ripening in all analyzed receptacles during fruit ripening. Also, ABA-glucose ester content increased during ripening in diploid *F.* × *vesca*, varieties, but decreased in octoploid *F.* × *ananassa*. In Chinese jujube, it was shown that *ZjCYP707A1* and *ZjCYP707A5* are two major members showing relatively high expression, and the expression of *ZjCYP707A2* dramatically increased at the onset of fruit ripening, whereas *ZjCYP707A5* expression appeared to be increased during fruit ripening. These observations indicate that *CYP707As* gene doesn’t act to promote ABA accumulation, because its expression was increased rather than decreased during fruit ripening. Given these observations, suppression of ABA catabolism is not likely to be the mechanism for triggering the HSP of ABA, although it may contribute to ABA accumulation during fruit ripening.

### IAA

IAA biosynthesis has been proposed to be mainly through the Trp-dependent pathway (Mano and Nemoto 2012). This pathway again includes four major pathways, i.e., the indole-3-acetamide (IAM) pathway; the indole-3-pyruvic acid (TAA/IPA/YUC) pathway; the tryptamine (TAM) pathway; and the indole-3-acetaldoxime (IAOX) pathway, among which the IPA pathway is believed to be the major pathway controlling IAA biosynthesis (Zhao 2010; Mano and Nemoto 2012; Blakeslee et al. 2019; Cao et al. 2020). While there may exist many potential rate-limiting steps in IAA biosynthesis pathways, only YUCs, which function as flavin-containing monooxygenases (FMO) catalyzing the conversion of IAA from the irreversible oxidative decarboxylation of indole-3-pyruvate acid (IPyA), has been currently thought to be the most important rate-limiting enzyme (Casanova-Sáez et al. 2021; Solanki and Shukla 2023).

IAA biosynthesis is believed to be triggered by fertilization whereby promoting fruit set (Figueiredo et al. 2015; An et al. 2020; Guo et al. 2022). Consequently, IAA plays a critical role in the regulation of the early FF development. As a negative signal, IAA production is decreased during FF ripening. TAA/TAR and YUCCA proteins have been demonstrated to jointly control IAA biosynthesis (Gallavotti et al. 2008; LeCLere et al. 2010; Abu-Zaitoon et al. 2012; Bernardi et al. 2012; Pattison et al. 2014). A few of studies reported the expression of *YUCCA* along with FF development and ripening. In *Fragaria vesca* L. genome, Liu et al. (Liu et al. 2012) identified two *YUC* genes, *FaYUC1* and *FaYUC2*, and they showed that both *FaYUC1* and *FaYUC2* transcripts were not detectable in the ripening receptacle, while a moderate expression of them was detectable in the green stages. Further, it was shown that *FaYUC1* and *FaYUC2* were highly expressed in the achenes, whereas *FaYUC1* transcript was increased at the onset of fruit ripening (Liu et al. 2012). Another study identified three IAA biosynthesis genes, i.e., *FaTAA1*, *FaTAR1*, and *FaTAR2*; and five *YUC* genes, i.e., *FaYUC2*, *FaYUC4*, *FaYUC7*, *FaYUC10*, and *FaYUC1* and it was shown that the transcripts of *FaTAA1*, *FaTAR2* and *FaTAR2* were substantially decreased throughout achenes’ development and ripening, implying that *FaTAA1*, *FaTAR2* and *FaTAR2* might act to regulate the level of IAA in the achenes, but not in the receptacle (Estrada-Johnson et al. 2017). Regarding *FaYUC*, it was shown that, except for *FaYUC10* that showed a decreased pattern in receptacle development and ripening, other *FaYUC* genes remained unchanged in their transcript level in both achenes and receptacle development and ripening, implying that *FaYUC* genes are not likely the determinator of the decreased level of IAA during FF ripening (Estrada-Johnson et al. 2017).

In peach (*Prunus persica* L. Batsch) genome, Pan et al. (Pan et al. 2015) identified 11 members of *PpYUC*, and they showed that nearly all of their transcripts were not detectable in the fruit flesh, although *PpYUC10/11* showed a low expression at the ripening stage. In contrast, more than half of the *PpYUC* genes (*PpYUC1/4/6/8/9/10/11*) were found to be highly expressed in the seed tissue. Specifically, while *PpYUC6/9/11* were highly expressed in the green stages, *PpYUC1/4/8/10* were highly expressed during fruit ripening. Overall, these observations suggest that the regulation of *PpYUC* expression in flesh is not likely to be critical to trigger the decrease of IAA level during fruit ripening. Given the observation that *YUC* genes were mainly expressed in achenes, the IAA export from achenes may play a more important role in the regulation of the IAA level in flesh.

## Initiation of HSP determined by hormonal transport

Hormonal transport includes export out of or import into specific organs/tissues, consequently leading to a change of the hormonal levels in specific organs/tissues. As thus, it may potentially play an important role in HSP in FF development and ripening. Hormonal transport has been reviewed several times, but largely focusing on its molecular bases (Boot et al. 2016; Hu and Shani 2023; Zhang et al. 2023c; Akhiyarova et al. 2024). Below is a summary of the updated knowledge on the hormonal transport in relation to HSP with the molecular basses not included. Because less information is available about the hormonal transport in relation to HSP in FF ripening, some knowledges on the common biological function of the hormonal transport is presented with the aim of providing valuable information on the potential role of the hormonal transport in HSP in FF development and ripening.

### Ethylene

Ethylene (ET) is a gaseous hormone, therefore, its transport can be simply achieved by diffusion, and this is just consistent with the fact that there is no ET transporter identified so far. Nevertheless, ET can be indirectly transported via ACC (Shin et al. 2015; Pattyn et al. 2021; Zhang et al. 2023c). There is evidence for the presence of ACC transporters (Hirner et al. 2006; Choi et al. 2019). For example, an early study by Bradford and Yang (Bradford and Yang 1980) reported that ACC levels in both roots and xylem sap increased markedly in response to waterlogging or root anaerobiosis in tomato plants. Also, ACC transport was reported to occur from root to shoot in sunflower (*Helianthus annuus* L.) seedlings (Finlayson et al. 1991). A study by Tudela and Primo-Millo (Tudela and Primo-Millo 1992) showed that water stress promoted ACC synthesis in the roots of *Cleopatra mandarin* seedlings and rehydration of the plants resulted in ACC transport to the shoots, where it was oxidized to ethylene. Subsequently, this ethylene induces leaf abscission. Moreover, there is a report that ACC transport may act to mediate plant growth under low-potassium nutrition in tomatoes (Martínez-Andújar et al. 2016). These observations suggest that ACC transport is potentially involved in ET signaling in many biological processes (Zhang et al. 2023c). Unfortunately, no information is available about relevance of ethylene transport with FF ripening.

### ABA

Theoretically, ABA transport can be achieved either through passive diffusion or a positive transport mechanism. ABA transporters were identified in *Arabidopsis* more than ten years ago (Kang et al. 2015; Kuromori et al. 2010), implying the presence of ABA active transport. A number of studies demonstrated that ABA transport may play an important role in some biological processes. In *Arabidopsis*, for example, a study by Kang et al. (Kang et al. 2015) identified ABA transporters as ATP-binding cassette (ABC) G-family proteins, designated as ABCG, and it was shown that ABCG25 and ABCG31 acted to transport ABA out of the endosperm and that ABCG30 and ABCG40 acted to transport ABA into the embryo, thus promoting seed dormancy. MtABCG20 was an ABA exporter and shown to be implicated in the regulation of lateral root and nodule development (Pawela et al. 2019). Also, ABCG17 and ABCG18 were proposed to be involved in the regulation of stomatal movement (Anfang and Shani 2021). In rice, OsDG1 was identified as another type of ABA transporter, and was proposed to regulate grain filling and lateral root emergence (Qin et al. 2021).

In nature, fruit production of plants is intrinsic to seeds’ dispersal, so that plants can survive and thrive in an ever-changing environment (Fukano and Tachiki 2021). Accordingly, the progress of seeds’ development and maturation should be orchestrated with that of the whole fruit, which implies a potential involvement of hormonal signaling communication between seed and flesh. ABA is well known to be critical to seed development and maturation, implying a potential involvement of ABA transport between seeds and flesh.

In strawberry, the pattern of ABA distribution between achenes and receptacle varies very much among studies. For example, Gu et al. (Gu et al. 2019) showed that the ABA content is several times higher in receptacle than in achenes during fruit ripening. Also, Figueroa et al. (Figueroa et al. 2021) reported the concentration of ABA and its metabolites in the receptacle was generally 100 times higher than in achenes. By contrast, Symons et al. (Symons et al. 2012) reported that ABA content was much higher in achenes than in receptacle during fruit ripening. Recently, a study by Li et al. (Li et al. 2022b) reported that the content of ABA in the achenes was basically identical to that in the receptacle before the onset of fruit ripening. In bill berry, the ABA content in berry tissue was also reported to be much higher than in seeds at the fully ripe stage (Karppinen et al. 2013). Despite these observations, it remains unclear whether ABA may be able to transport between seeds and fruit flesh.

### IAA

Among the three hormones, IAA is the only one that has been well demonstrated to play a critical role in both FF development and ripening via transport regulation. By using reporter genes, Feng et al. (Feng et al. 2019) demonstrated that the genes for IAA biosynthesis were primarily expressed in the endosperm and embryo inside the achenes. Consistent with this, the level of IAA is much higher in seeds than in flesh. In strawberry, for example, it was reported that the level of IAA might be several tens of times higher in the achenes than in the receptacle (Symons et al. 2012; Estrada-Johnson et al. 2017; Gu et al. 2019). Similarly, in other fruits, such as grape berry (Gouthu and Deluc 2015; Tian et al. 2022), tomato (Hocher et al. 1992) and apple (Devoghalaere et al. 2012), the IAA content in seeds was all reported to be several tens of time higher than in the flesh. These studies suggest that IAA was primarily produced in seeds. As a growth promoting hormone, on one hand, IAA should act to promote flesh growth and expansion, and on the other hand, it acts to suppress fruit ripening. Consistent with this theory, a study by Li et al. (Li et al. 2022b) showed that removal of achenes resulted in suppression of receptacle growth and expansion and promotion of coloration, demonstrating the importance of IAA transport in the control of both fruit growth and ripening. As a negative signal of fruit ripening, fruit ripening is premised on a substantial decrease in the level of IAA in the flesh, but it remains unclear whether a decreased export of IAA out of seeds was induced during fruit ripening, thereby relieving its suppression on fruit ripening.

## Interplay of hormonal biosynthesis in relation to HSP

**Fig. 3:**
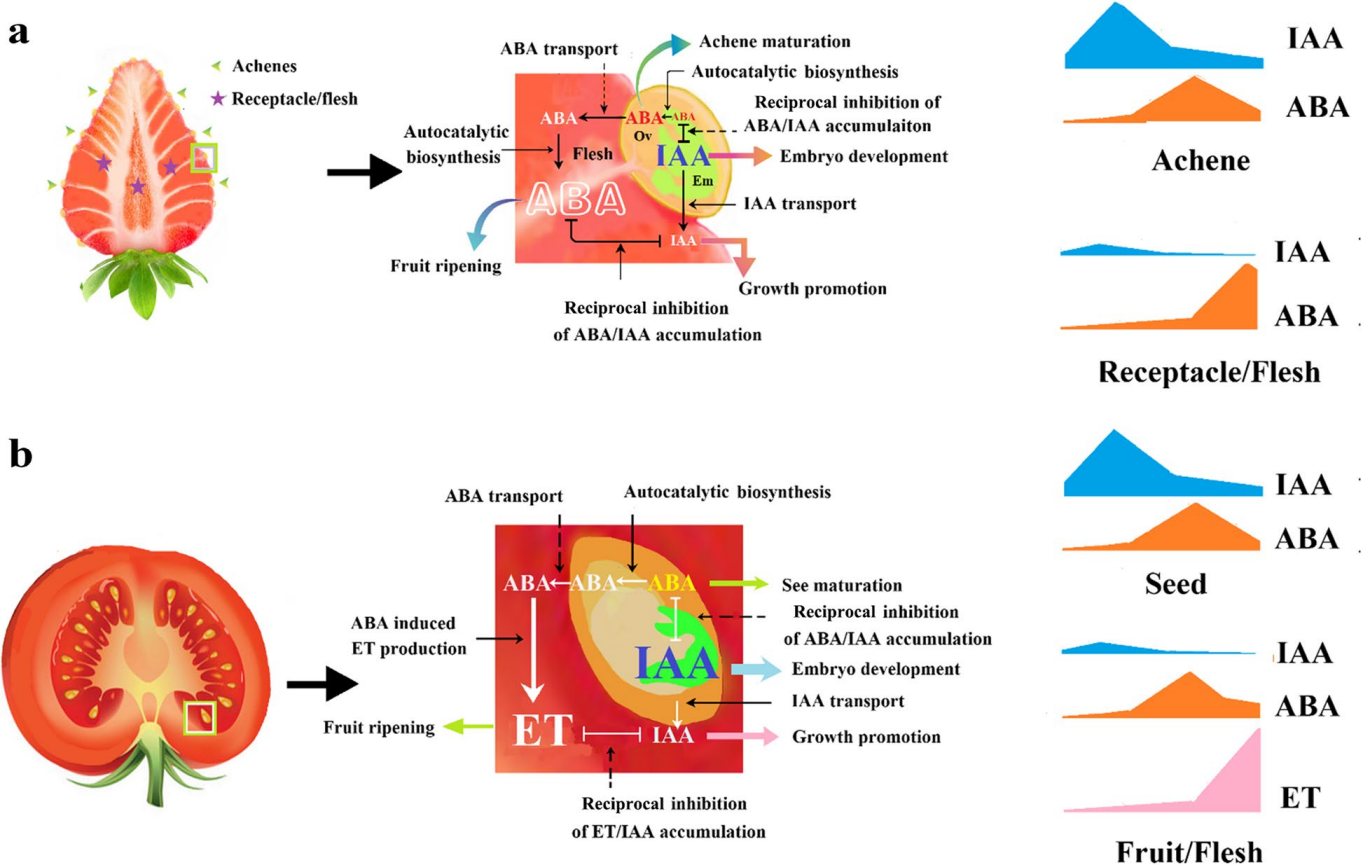
Diagram showing the hormonal function in relation to their communication. **a** the left, whole fruit with strawberry as a model of NC fruit, showing the structural relationship between achenes/seeds and receptacle/flesh; the middle, amplification of the part in boxed in green in the whole fruit, showing the pattern of hormonal transport in relation to their signal production; the right, showing the changing patterns in the levels of IAA and ABA from fruit set to ripening. **b** the left, the whole fruit with tomato as a model of CL fruit, showing the structural relationship between achenes/seeds and receptacle/flesh; the middle, amplification of the part in boxed in green in the whole fruit, showing the pattern of hormonal transport in relation to their signal production; the right, showing the changing patterns in the levels of IAA, ABA and ET from fruit set to ripening

Interplay of hormonal biosynthesis refers to that one hormone acts to modulate the biosynthesis of another hormone. Additionally, biosynthesis of a hormone might be regulated by the hormone itself, i.e., a feedback regulation of the hormonal biosynthesis, leading to the signal amplification of the hormone (Fig. 3). Unveiling the mechanism for the interplay and feedback regulation of hormonal biosynthesis is crucial to understanding the mechanism of HSP and how multiple hormones collaboratively regulate fruit development and ripening.

### Involvement of ABA induced ET production in FF ripening

As early as in the 1970s, it was reported that dehydrated leaves of Valencia orange induced ethylene accumulation (Ben-Yehoshua and Aloni 1974). Moreover, it was reported that ABA was able to induce callus formation in cultured buds of Shamouti orange (*Citrus sinensis* L. Osbeck) via modulation of ethylene production (Goren et al. 1979). Also, it was demonstrated that exogenous ABA significantly stimulated ethylene production in citrus (*Citrus sinensis* L. Osbeck, cv. Shamouti orange) leaf discs. There is evidence that ABA-induced ET production is involved in FF ripening. For example, a study by Jiang et al. (Jiang et al. 2000) demonstrated that exogenous ABA was able to induce banana fruit ripening and ethylene production, whereas ethylene action inhibitor, 1-methylcyclopropene (1-MCP), suppressed ABA induced fruit ripening. Mou et al. (Mou et al. 2016) reported that exogenous ABA was able to induce tomato fruit ripening and facilitate ethylene production and 1-MCP suppressed ABA induced tomato fruit ripening and ethylene production. Also, it was reported that the expression of *LeNCED1*, a key gene for ABA biosynthesis, just peaked before the promotion of the key genes for ethylene biosynthesis (such as *LeACS2*, *LeACS4*, and *LeACO1*) (Zhang et al. 2009b). These observations suggest that ABA may function as an early signal to regulate CL fruit ripening via modulating ET production.

### Reciprocal regulation of hormonal biosynthesis between ABA and IAA

In *Arabidopsis*, accumulating evidences suggest that auxin and ABA may jointly act to regulate various processes, including seed germination (Monroe-Augustus et al. 2003; Belin et al. 2009; Liu et al. 2013; Thole et al. 2014), hypocotyl and root elongation (Monroe-Augustus et al. 2003; Thole et al. 2014; Lorrai et al. 2018; Emenecker and Strader 2020; Emenecker et al. 2023; Qin et al. 2023), and lateral root formation (Shin et al. 2007; Shkolnik-Inbar and Bar-Zvi 2010; Xing et al. 2016; Lu et al. 2019), etc. Hypocotyl elongation was inhibited by IAA, whereas mutants defective in ABA biosynthesis was insensitive in their hypocotyl elongation to IAA, implying that regulation of ABA biosynthesis was involved in IAA action on hypocotyl elongation (Emenecker et al. 2023). A study by Lei et al. (Lei et al. 2023) reported that the expression of an IAA oxidase enzyme, *SlDAO2*, was induced by both external and internal ABA in tomato hypocotyls. Furthermore, an ABA-responsive regulatory *SlAREB1/SlABI3-1/SlABI5* cascade was identified to act upstream of *SlDAO2* and to precisely control its expression, suggesting that ABA may act to suppress IAA accumulation in vegetative organs/tissues.

It has been increasingly suggested that reciprocal regulation of HSP between ABA and IAA is also involved in the regulation of FF development and ripening. In tomato, for example, the ABA coreceptor, type 2C protein phosphatase (SlPP2C2), could interact with flavin monooxygenase FZY, an indole-3-acetic acid (IAA) biosynthetic enzyme, whereby activating FZY activity, implying that ABA could inhibit IAA biosynthesis (Li et al. 2024). In peach, it was reported that application of exogenous NAA, analog of IAA application, markedly increased IAA content and drastically decreased ABA content at the fruit expansion stage. Consistent with this, NAA application was found to promote the expression auxin biosynthesis gene (*PpYUCCA*) and early auxin-responsive genes (*PpIAA*, *PpGH3*, *and PpSAUR*), and inhibit the ABA biosynthesis genes (*PpNCED*, *PpABA3*, and *PpAAO3*) (Zhang et al. 2023b). In strawberry, Liao et al. (Liao et al. 2018) reported that IAA could suppress the gene expression of *FveCYP707A4a*, a key enzyme catalyzing ABA catabolism. As thus, the decreased level of IAA during fruit ripening would be expected to promote ABA accumulation, and accordingly, it was proposed that it was the decrease of the IAA level that acts to initiate NC fruit ripening.

### Reciprocal regulation of hormonal biosynthesis between ethylene and IAA

There is evidence that the HSP of IAA and ET is reciprocally regulated. Several decades ago, a number of studies reported that IAA was able to induce ET production in pea (*Pisum sativum*). IAA-induced ET production was involved in diverse processes, such as suppression of root growth (Andreae et al. 1968; Chadwick and Burg 1970), bud development (Burg and Burg 1968) and development of shoot epidermal cells (Sargent et al. 1974). IAA was reported to be able to promote ACS activity and *ACO* expression (Peck and Kende 1995). The pattern of IAA-induced ET production varies among plant species and organs/tissues. In *Arabidopsis *, IAA-induced ET production occurs mainly in the youngest leaves and root tips (Arteca and Arteca 2008). Additionally, it is reported that IAA-induced ET production may involve ABA production. For example, IAA was reported to induce the expression of *GaNCED1*, a key gene of ABA biosynthesis in *Galium aparine*, proceeded with ET production (Kraft et al. 2007). In tomato, both ethylene and ABA production could be stimulated by IAA in the shoot tissue, whereas in tomato mutants defective in ethylene perception (e.g. never ripe), IAA did not induce ABA accumulation (Hansen and Grossmann 2000). In peach, overexpressing *PpILR1*, a gene encoding IAA-amino hydrolase, promoted ET production and fruit ripening (Wang et al. 2021a). Tatsuki et al. (Tatsuki et al. 2013) compared the pattern of IAA induced ET production in two peach cultivars with different fruit texture, melting flesh peaches and stony hard peaches, and found that IAA concentration increased just before harvest time in melting flesh peaches, coinciding with system II ethylene production, whereas did not increase in stony hard peaches. Accordingly, it was proposed that a high concentration of IAA was required to generate system II ethylene (Kuromori et al.  2010).

In apple, Devoghalaere et al. (Devoghalaere et al. 2012) observed that IAA levels dramatically increased in seeds, whereas remained unchanged in the cortex. Also, in tomato, IAA levels were found to be substantially increased during seed maturation, and as the seed matured, decreased (Hocher et al. 1992). Pattison and Catalá (Pattison and Catalá 2012) investigated the changing pattern of IAA levels and distribution in different fruit tissues, including seeds, placental tissue and the central columella, and found that the IAA levels were high in all the tissues at the early developmental stage (e.g. 5 DPA), then decreased during the following growth stage (e.g. 21 DPA). Strikingly, when the fruit had reached its final size at 34 DPA, levels of IAA remarkably increased in all tissues, especially in seeds, then declined to the levels undetectable during fruit ripening. IAA is a growth-promoting hormone, so it is not reasonable to propose that IAA acts to trigger ET production, otherwise, application of IAA would expect to promote rather than suppress fruit ripening.

There are reports that IAA catabolism could be induced by ET. In apple, for example, the gene expression of *MdGH3*, which acts to remove free auxin by conjugation to amino acids, dramatically increased at the onset of fruit ripening (Devoghalaere et al. 2012; Kumar et al. 2012). Moreover, *GH3* expression could be induced by ET in many CL fruit species, for example, in *Capsicum chinense* L. (Liu et al. 2005), and in tomato (Sravankumar et al. 2018), indicating that ET acts to suppress IAA accumulation via activation of GH3. In addition to IAA catabolism, IAA transport may be modulated by ET. As early as 1966, Morgan and Gausman (Morgan and Gausman 1966) reported that ET was capable of inhibiting IAA transport in petiole tissue of cotton. Also, in etiolated pea epicotyls, basipetal IAA transport was reported to be inhibited by ET (Suttle et al. 1988). In *Arabidopsis*, genetic analysis demonstrated the effect of ET on IAA transport, as evidenced by the observation that *aux1*, *lax3*, *pin3* and *pin7*, which are defective in auxin influx and efflux proteins, were less sensitive to the inhibition of lateral root formation and stimulation of auxin transport in response to ACC treatment (Lewis et al. 2011). It remains unclear whether the burst of ET during fruit ripening may act to inhibit IAA transport from seeds to flesh, whereby leading a decrease in the IAA levels. Overall, it is likely the burst of ethylene may act to cause an IAA decrease in flesh via promoting IAA conjugation or inhibiting IAA export from seeds.

## Feedback regulation of hormonal biosynthesis in relation to hormonal signal amplification

### Ethylene signal amplification

McMurchie et al. (McMurchie et al. 1972) postulated the involvement of two ET production systems in CL fruit development and ripening, i.e., system I, which is functional during normal vegetative growth, and System II, which functions to trigger fruit ripening. It is believed that system I ET acts to trigger system II ET, implying that the production of system II ET is an autocatalytic process, or, positive feedback regulation of biosynthesis (Bufler 1986; Blume and Grierson 1997; Bouquin et al. 1997; Lelièvre et al. 1997; Nakatsuka et al. 1998). While a coordinated action of system I and system II has been proposed to be involved in the regulation of fruit development and ripening, it remains unclear how system I ET transition is converted into system II ET. To understand the relationship between system I and system II ET, a study by Yokotani et al. (Yokotani et al. 2009) compared the pattern of ET production in the wild type tomato with that in the *RiEIL* tomato defective in ET signaling due to RNAi inhibition of the expression of *LeEIL*, a key component in ET signal production. While the ET sensitivity was largely suppressed in *RiEIL* tomato, it failed to completely block ET production. Furthermore, 1-methylcyclopropene (1-MCP), a potent inhibitor of ethylene perception, also failed to inhibit the increase in ET production in *RiEIL* tomato, accordingly, the authors proposed that the system II may consists of two parts: a small part regulated by a developmental factor and a large part regulated by an autocatalytic system. Additionally, even if the effect of system I ET is eliminated, fruit still showed a small increase in ET production, therefore, it was proposed that system I was less likely to be involved in the transition to system II.

Although autocatalytic ET production is believed to be triggered by a developmental factor, little is known about the nature of this developmental factor (Yokotani et al. 2009). In nature, fruit production is motivated to disperse seeds, accordingly, seeds and fruit development and ripening are highly coordinated. Specifically, fruit ripening is premised on seed maturation, and once seed maturates, it will initiate the ripening of whole fruit to facilitate seeds dispersal. As thus, the seed-to-flesh signaling may serve as a development factor influencing the HSP of ET. Since seeds’ maturation is accompanied with a dramatic increase in both IAA and ABA, it is of great significance and importance to clarify whether seeds’ maturation-associated IAA and ABA may act to trigger HSP of ET.

### ABA signal amplification

Accumulating studies suggest the existence of autocatalytic ABA production and its involvement in diverse biological processes. In *Arabidopsis*, for example, a few studies suggest that expression of the ABA biosynthetic genes, such as *NCED3*, *AAO3*, *ABA1* and *ABA3*, was induced by exogenous ABA (Xiong et al. 2002; Barrero et al. 2006). In peanut plants (*Arachis hypogaea*), the expression of *AhNCED1* was induced by exogenous ABA (Wan and Li 2006). Recently, Yang and Tan (Yang and Tan 2014) identified the presence of ABA responsive element in the promoter of *AtNCED3*, suggesting the involvement of catalytic ABA biosynthesis in vegetative growth. In recent years, autocatalytic ABA production has been increasingly demonstrated to be involved in fruit development and ripening. In strawberry, for example, a study by Liao et al. (Liao et al. 2018) reported that ABA could repress the expression of *FveCYP707A4a* and promote the expression of *FveNCED*. In peaches, Tian et al. (Tian et al. 2020) demonstrated that exogenous ABA was capable of enhancing the activities of a series of ABA biosynthesis, including aldehyde oxidase (AO), 9-*cis*-epoxycarotenoid dioxygenase (NCED), and zeaxanthin epoxidase (ZEP) of ABA synthesis during cold storage, and upregulated the gene expression of *PpAO1*, *PpNCED1*, *PpNCED2*, and *PpZEP*. These observations suggest a positive feedback regulation of ABA production in fruits. So far, little is known about the triggering mechanism of the autocatalytic ABA production in fruits. It has been well established that seeds’ maturation is accompanied with a massive ABA accumulation whereby avoiding seed germination before harvest. The ABA accumulation in seeds may be a potential signal to trigger ABA autocatalytic production, leading to a massive ABA accumulation in the flesh. Consistent with this notion, Li et al. (Li et al. 2022b) demonstrated that autocatalytic ABA production largely occurred in achenes, suggesting the importance of autocatalytic ABA production in seeds. A comprehensive study on the spatiotemporal pattern of ABA accumulation within different tissues is critical to reveal the initial signal triggering ABA autocatalytic production.

## Signal transduction of HSP for ET and ABA

**Fig. 4:**
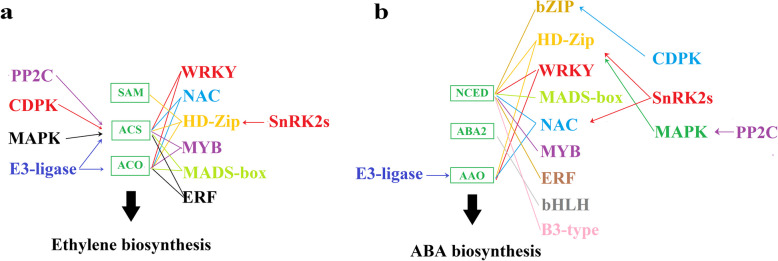
Diagram showing the signaling network upstream of ethylene and ABA production. **a** the signaling network implicated in the regulation of ethylene biosynthesis; **b** the signaling network implicated in the regulation of ABA biosynthesis. Different cascades are shown in different colors. Note that the key enzymes can be regulated both transcriptionally and post-transcriptionally, and that the transcriptional regulation is determined by the transcription factors, which are again modulated by the upstream signaling proteins, and the post-transcriptional regulation is directly modulated by the upstream signaling proteins

In terms of the physiological and biochemical bases, HSP is determined by the functional proteins controlling hormonal metabolism and transport, whereas in terms of the mechanism of signal transduction, HSP is determined by the signal proteins upstream of the functional proteins, which constitute a complex of signaling network to modulate the activity of the functional proteins and transcription factors (Fig. 4). To understand the mechanism of HSP, thoroughly deciphering the picture of the signaling network is required. ET and ABA are two major positive signals directly triggering fruit ripening, whereas IAA acts to control fruit growth and expansion and antagonize the action of ET and ABA. Consequently, IAA is excluded from the subsequent summary and discussion.

### Ethylene

Studies on ET production have been largely focusing on the identification and functional characterization of the transcription factors that act to regulate the gene expression of *ACO* and *ACS*. So far, a large number of transcription factors have been identified that act to regulate the expression of *ACS* and *ACO*. These transcription factors belong to diverse gene families, such as the Homeodomain Leucine Zipper (HD-Zip), the basic region leucine zipper (b-Zip); WRKYGQK (hence called WRKY), NAC, MADS box (Table 2). Some transcription factors act to regulate *ACS* expression (Hu et al. 2018; Yue et al. 2020; Nieuwenhuizen et al. 2021; Wang et al. 2022b; Wang et al. 2023b), whereas the others act regulate *ACO* expression (Manavella et al. 2006; Zheng et al. 2020; Wang et al. 2021b; Wang et al. 2023b). In some cases, several transcription factors may form complex to collaboratively regulate *ACO* or *ACS* expression, whereas, in some other cases, a transcription factor may act to regulate the expression of another transcription factor, which again regulates the expression of *ACS* or *ACO* (For details, please see the summary in Table 2). The involvement of diverse transcription factors in the regulation of ACS and ACO implies that the HSP of ET is determined by a complex of internal and external cues.

**Table 2:** The signaling components mediating ethylene production

Species/Latin name	Target genes	TF name	TF family	Function	Reference
Sunflower/*Helianthus Annuus*	*ACO*, *SAM*	Hahb-4	HD-Zip	Downregulation	(Manavella et al. 2006)
Litchi/*Litchi chinensis* Sonn	*LcACO2/3*,* LcACS1/4/7*	LcHB2, LcHB3	HD-ZIP	Upregulation	(Li et al. 2019)
Wheat/*Triticum aestivum* L	*TaACS2*, *TaACS7*,* TaACS8*	TaWRKY51	WRKY	Downregulation	(Hu et al. 2018)
*Arabidopsis thaliana*	*ACS5/6/8/11*,* ACO5*	AtWRKY29	WRKY	Upregulation	(Wang et al. 2023b)
*Arabidopsis thaliana*	*ACS5/11*,* ACO5*	AtWRKY22	WRKY	Upregulation	(Wang et al. 2024)
*Solanum habrochaites*	*ShACO*, *ShACS*	ShNAC1	NAC	Upregulation	(Liu et al. 2018)
Kiwifruit/*Actinidia chinensis*	*AcACO1*	AcNAC3, AcNAC4	NAC	Upregulation	(Fu et al. 2024)
Kiwifruit/*Actinidia chinensis*	*AcACS1*	AcNAC1-4	NAC	Upregulation	(Nieuwenhuizen et al. 2021)
*Arabidopsis thaliana*	*ACS*	MYB102	MYB	Upregulation	(Zhu et al. 2018)
Wheat/*Triticum aestivum*	*TuACO3*	AtMYB46	MYB	Upregulation	(Zheng et al. 2020)
Apple/*Malus domestica*	*MdACS3a*,* MdACS1*	MdARF5, MdERF2	ARF, ERF	Upregulation	(Yue et al. 2020)
Tomato/*Lycopersicon esculentum*	*LeACS2*, *LeACS4*, *LeACO1*,* LeACS6*	RIN	MADS box	Upregulation	(Li et al. 2011)
Apple/*Malus domestica*	*MdACS1*	MdERF2	ERF	Downregulation	(Li et al. 2016)
Tomato and Tobacco	*NtACS3*,* LeACO3*	*Le*ERF2/TERF2	ERF	Upregulation	(Zhang et al. 2009c)
Kiwifruit/*Actinidia deliciosa*	*AdACS1/2*,* AdACO5*	AdERF105L and AdWRKY29	ERF&WRKY	Downregulation	(Wang et al. 2022b)
Kiwifruit/*Actinidia deliciosa*	*AdACS1*	AdNAC2/3	NAC	Upregulation	(Wang et al. 2022b)
Peach/*Prunus persica*	*PpACO1*	PpIAA1-PpERF4	Aux/IAA, ERF	Upregulation	(Wang et al. 2021b)
Plums/*Prunus salicina*	*PsACS1*	PsABI5, PsGL2, PsTCP2	b-Zip, HD-ZIP IV, TCP	Upregulation	(Sadka et al. 2019)
Species/Latin name	TF or enzymes	Signal proteins or modules		Function	Reference
Apple/*Malus domestica*	MdACO1-MdHB1	MdSnRK2.4, MdSnRK2.9		MdHB1 activity via phosphorylation	(Jia et al. 2022)
Tomato/*Solanum lycopersicum*	SlACS2, SlACS4	SlMAPK3		Stability regulation via Phosphorylation	(Shu et al. 2023)
Banana/*Musa acuminata*	Ma-ACS1	Ser/Thr-Protein kinase		Stability regulation via Phosphorylation	(Choudhury et al. 2012)
Kiwifruit/*Actinidia deliciosa*	AdACS3	AdMPK16		Stability regulation via phosphorylation	(Wang et al. 2022b)
Tobacco/*Nicotiana tabacum*	NtACS, NtACO	NtMEK2-SIPK/WIPK		Activity regulation via phosphorylation	(Kim et al. 2003)
Cotton/*Gossypium hirsutum*	GhACS4, GhACO1	GhXB38D		E3 ubiquitin ligase mediated degradation	(Song et al. 2023)
*Arabidopsis thaliana*	ACS2, ACS6	MPK6		Stability regulation via Phosphorylation	(Liu and Zhang 2004)
Tomato/*Lycopersicon esculentum*	*Le*ACS2-Ser460	*Le*CDPK2		Stability regulation via Phosphorylation	(Hernández Sebastià et al. 2004)
*Arabidopsis thaliana*	ACS2/ACS6	MPK6		Stability regulation via Phosphorylation	(Joo et al. 2008)
Tomato/*Lycopersicon esculentum*	LeACS2	LeCDPK2		Stability regulation via Phosphorylation	(Kamiyoshihara et al. 2010)
*Arabidopsis thaliana*	ACS2/ACS6	MPK3/6		Stability regulation	(Han et al. 2010)
*Arabidopsis thaliana*	CS2 and ACS6	MPK3/6-ERF1A		Stability regulation via phosphorylation	(Wang et al. 2022d)
*Arabidopsis thaliana*	ACS2/6/7/8	CPK5, CPK6		Stability regulation via phosphorylation	(Li et al. 2018)
*Arabidopsis thaliana*	ACS2/6/8	MKK4/6-MPK3/6		Stability regulation	(Li et al. 2018)
*Arabidopsis thaliana*	ACS2/6	MPK3/6-WRKY33		Transcription regulation via TF phosphorylation	(Li et al. 2012)
*Arabidopsis thaliana*	ACS6	PP2A		Stability regulation via dephosphorylation	(Skottke et al. 2011)
*Arabidopsis thaliana*	ACS6	ABI1		Stability phosphorylation via dephosphorylation	(Ludwików et al. 2014)

The table above the dashed line shows the key enzymes as well as their upstream transcription factors involved in ethylene biosynthesis; the table below the dashed line shows the signal proteins upstream of the transcription factors or the key enzymes involved in ethylene biosynthesis. Note that the key enzymes can be regulated both transcriptionally and post-transcriptionally

Signal transduction is commonly achieved by the protein modification, in which protein kinase/phosphatase catalyzed reversible protein phosphorylation is believed to be the major mechanism of protein modification. While a large number of transcription factors have been identified, much less is known about the signaling mechanism upstream of the transcription factors. To date, only a limited protein kinases and phosphatases have been reported to be implicated in the regulation of ET production. Strikingly, most of the studies have been focusing the MAPK (Mitogen Activated Protein Kinase) signaling implicated in the transcription or post-transcription regulation of ACS or ACO. Notably, among the MAPKs, MPK3 and MPK6 have been a focus of research. For example, in tobacco, Kim et al. (Kim et al. 2003) reported that over expression of *SIPK/WIPK*, a MAPK, was able to induce ET accumulation, which coincided with a dramatic increase of the ACC and ACS activities. Subsequently, it was demonstrated that unphosphorylated ACS6 protein was rapidly degraded by the 26S proteasome pathway and the MAPK catalyzed ACS6 phosphorylation acts to stabilize the ACS6 protein (Joo et al. 2008). Similarly, Liu and Zhang (Liu and Zhang 2004) demonstrated that MPK6, the *Arabidopsis thaliana* ortholog of tobacco SIPK, was able to stabilize ACS2 and ACS6 via phosphorylation, leading to the accumulation of ACS protein and ET accumulation. Besides MPK6, MPK3 was also able to phosphorylate ACS2/6 to modulate ET production (Han et al. 2010). Recently, it was reported that MPK3/MPK6 act to mediate negative feedback regulation of pathogen-induced ethylene production (Wang et al. 2022d), and in tomato, it was reported that SlMAPK3 was able to mediate cold-induced ethylene production (Shu et al. 2023).

Aside from MAPKs, CDPKs (Calcium Dependent Protein Kinase) have been also reported to be involved in the regulation of ET production. For example, it was reported that LeACS2 was readily phosphorylated in vitro by several CDPKs, suggesting the involvement of CDPK in the regulation of ET production in tomato fruit (Hernández-Sebastià et al. 2004). A calcium-dependent protein kinase (CDPK), LeCDPK2, was isolated as one of the protein kinases that are able to phosphorylate LeACS2 at Ser-460 and the phosphorylation/dephosphorylation of LeACS2 regulates its turnover upstream of the ubiquitin-26S-proteasome degradation pathway (Kamiyoshihara et al. 2010). Wounding is well known to induce ET production. In *Arabidopsis*, a few of studies showed that wounding-induced ET production was mediated by both MAPK and CDPK signaling. Specifically, in the MAPK pathway, ET production was mediated by MKK4/5- MPK3/6 signaling module, whereas, in the CDPK pathway, it was mediated by CPK5/6 via regulating the expression of *ACS2*, *ACS6* and *ACS8* genes (Li et al. 2018).

Besides protein kinase, protein phosphatase was also reported to play an important role in ET production. For example, Skottke et al. (Skottke et al. 2011) demonstrated that PP2A-deficient plants caused ethylene overproduction and that RCN1-containing PP2A complexes specifically dephosphorylated a C-terminal ACS6 phosphopeptide. Similarly, Ludwików et al. (Ludwików et al. 2014) reported that *Arabidopsis* protein phosphatase 2C ABI1 interacted with ACS6 and dephosphorylated its C-terminal fragment of MPK6 to affect the ACS6 stability. Li et al. (Li et al. 2012) reported that WRKY33, a transcription factor binding to the W-boxes in the promoters of ACS2 and ACS6, could be phosphorylated by MPK3/MPK6, implying the presence of the signaling module MPK3/MPK6-WRKY33-ACS2 and ACS6 in the regulation of ET production. Since a large number of transcription factors have been identified to control the expression of *ACS* and *ACO*, greater emphasis should be given to the study of the signaling cascades that occur previously to the transcription factors. An in-depth investigation of the MAPK or CDPK signaling pathway upstream of ACS and ACO might enhance our knowledge of the mechanism behind the HSP of ET.

### ABA

As mentioned above, while NCED has been commonly thought to be the rate-limiting enzyme in the ABA biosynthesis pathway, some other enzymes, such as ABA2 and AAO3, may potentially become the rate-limiting enzymes for the ABA accumulation under some conditions (Seo and Koshiba 2002). Consistent with this, a large number of transcription factors have been identified to bind to the promoters of *NCED*, as well as *ABA2* and *AAO3*, etc. These transcription factors belong to a diversity of gene families, including WRKY, MYB, NAC, HB, ERF, b-ZIP, etc. As summarized in Table 3, while most of the transcription factors function to enhance the expression of *NCED*, *ABA2* and *AAO3*, some transcription factors may inhibit their expression. A limited number of studies have reported some members of protein kinases and phosphatases involved in the regulation of ABA production, but mostly in *Arabidopsis* or crop plants, and less is known about their functions in fruit development and ripening. In *Arabidopsis*, Tan et al. (Tan et al. 2018) demonstrated that HAT1, a HD-ZIP transcription factor that acts to suppress *ABA3* and *NCED3* expression, could be phosphorylated by SnRK2s. In rice, Zong et al. (Zong et al. 2016) demonstrated that OsbZIP23, which acts to promote the expression of *OsNCED4*, could be phosphorylated by SAPK2, a homolog of SnRK2 protein kinase. In pepper (*Capsicum annuum* L.), Zhang et al. (Zhang et al. 2024b) demonstrated that CaNAC035, a NAC transcription factor that binds to the promoter of both *CaAAO3* and *CaNCED3*, could be phosphorylated by CaSnRK2.4. These observations suggest that SnRK2s play crucial roles in the regulation of ABA biosynthesis. Besides SnRK2s, CDPKs and MAPKs also have been reported to play important roles in the regulation of ABA biosynthesis. In wheat, for example, Zhang et al. (Zhang et al. 2023a) demonstrated that TabZIP60, a b-ZIP transcription factor that acts to promote *TaNCED2* expression, could be phosphorylated by TaCDPK30 at Ser110. Furthermore, TaCDPK30 could interact with protein phosphatase 2C clade A (TaPP2CA116/TaPP2CA121), thus making up a signaling module of TaPP2CA116/TaPP2CA121-TaCDPK30-TabZIP60-TaNCED2 functioning to regulate ABA biosynthesis.

**Table 3:** The signaling components mediating ABA production

Species/Latin name	Target genes	TF name	TF family	Function	Reference
Wheat/*Triticum aestivum* L	*TaNCED2s*	TaFDL2-1A	bZIP	Upregulation	(Wang et al. 2022a)
*Arabidopsis thaliana*	*ABA3*,* NCED3*	HAT1	HD-ZIP II	Downregulation	(Tan et al. 2018)
Litchi/*Litchi chinensis* Sonn	*LcNCED3*	HD-ZIP	HD-ZIP	Upregulation	(Li et al. 2019)
Citrus/*Citrus* spp.	*CsNCED2*	CsHB5-CsbZIP44	HD-ZIP-b-ZIP	Upregulation	(Sun et al. 2024)
Citrus/*Citrus* spp.	*NCED2*	CsHB5	HD-ZIP I	Upregulation	(Zhang et al. 2021)
Maize/*Zea mays* L	*ZmAAO3*	ZmWRKY79	WRKY	Induce	(Gulzar et al. 2021)
*Arabidopsis thaliana*	*MaNCEDs*	MaWRKY80	WRKY	Upregulation	(Liu et al. 2020)
*Tulipa gesneriana*	*TgNCED3*	TgWRKY75	WRKY	Upregulation	(Meng et al. 2024)
Maize/*Zea mays* L	*ZmNCED1*	ZmWRKY120	WRKY	Upregulation	(Jiao et al. 2024)
Carnation/*Dianthus caryophyllus* L	*DcNCED2*,* DcNCED5*	DcWRKY33	WRKY	Upregulation	(Wang et al. 2023a)
Cotton/*Gossypium hirsutum* L	*NCED2/5/6/9*	GhWRKY1	WRKY	Upregulation	(Hu et al. 2021)
Citrus/*Citrus* spp.	*CrNCED5*	CrNAC036—CrMYB68	NAC, MYB	Downregulation	(Zhu et al. 2020)
Peach/*Prunus persica*	*PpNCED2/3*	PpERF3	ERF	Upregulation	(Wang et al. 2019)
*Arabidopsis thaliana*	*NCED3*	NGATHA1	B3-type	Upregulation	(Sato et al. 2018)
*Gladiolus gandavensis* Vaniot Houtt	*GhNCED*	GhTCP19	TCP	Downregulation	(Wu et al. 2019)
*Arabidopsis thaliana*	*NCED2*,* NCED6*	MYB96	MYB	Upregulation	(Lee et al. 2015)
*Arabidopsis thaliana*	*AAO3*	NAP	NAC	Upregulation	(Yang et al. 2014a)
*Arabidopsis thaliana*	*NCED3*	ATAF1	NAC	Upregulation	(Jensen et al. 2013)
Tomato/*S. lycopersicum*	*SlNCED1*	SlNAP2	NAC	Upregulation	(Ma et al. 2018)
Rice/*Oryza sativa*	*OsNCED3*	OsNAC2	NAC	Upregulation	(Jiang et al. 2019)
Wheat/*Triticum aestivum* L	*AAO3*	TaNAC69-B	NAC	Upregulation	(Li et al. 2023)
Cotton/*Gossypium hirsutum* L	*GhNCED3a/3c*	GhirNAC2	NAC	Upregulation	(Shang et al. 2020)
Peanut/*Arachis hypogaea*	*AhNCED1*	AhAREB1-AhNAC2	b-ZIP-NAC	Downregulation	(Liu et al. 2016)
Wheat/*Triticum aestivum* L	*TaNCED3*	TaBZR1	BES1/BZR1	Upregulation	(Yang et al. 2023)
Cotton/*Gossypium hirsutum* L	*GhNCED1*	GhMYB102	R2R3-MYB	Upregulation	(Liu et al. 2023b)
Rice/*Oryza sativa*	*OsABA2*	OsbHLH110	bHLH	Upregulation	(Zhang et al. 2024a)
Pear/*Pyrus pyrifolia* Nakai	*PpNCED3*	PpAREB1-PpDAM1	MADS-box	Upregulation	(Tuan et al. 2017)
Yellowhorn/*Xanthoceras sorbifolium*	*XsNCED6* and* XsBG1*	XsAGL22	MADS-box	Upregulation	(Bian et al. 2022)
Species/Latin name	TF or enzymes	Signal proteins or modules	Function		Reference
*Arabidopsis thaliana*	NCED3	SnRK2.3-HAT1	Downregulation		(Tan et al. 2018)
Pepper/*Capsicum annuum* L	CaAAO3 and CaNCED3	CaSnRK2.4-CaNAC035	Upregulation		(Zhang et al. 2024b)
Rice/*Oryza sativa* L	Os-ABA2	OsMPK1-OsABA2	Stability		(Zhang et al. 2022)
Rice/*Oryza sativa* L	OsNCED3	miR2105-(OsSAPK10)-OsbZIP86	Upregulation		(Gao et al. 2022)
Wheat/*Triticum aestivum* L	TaNCED2	TaCDPK30- TabZIP60	Upregulation		(Zhang et al. 2023a)
Rice/*Oryza sativa* L	OsNCED4	OsPP2C49-SAPK2- OsbZIP23	Upregulation		(Zong et al. 2016)
*Arabidopsis thaliana*	AAO3	SAUL1-E3	E3-ligase mediated degradation		(Raab et al. 2009)

The table above the dashed line shows the key enzymes as well as their upstream transcription factors involved in ABA biosynthesis; the table below the dashed line shows the signal proteins upstream of the transcription factors or the key enzymes involved in ABA biosynthesis. Note that the key enzymes can be regulated both transcriptionally and post-transcriptionally

There is evidence that MAPK signaling plays an important role in the regulation of ABA biosynthesis. In rice, for example, a study by Gao et al. (Gao et al. 2022) demonstrated that OsbZIP86, a b-ZIP transcription factor that acts to enhance *OsNCED3* expression, could be phosphorylated by MAPK, OsSAPK10. Moreover, OsbZIP86 was also shown to be regulated by miR2105-directed cleavage of the OsbZIP86 mRNA, thus constituting a signaling module of miR2105/ OsSAPK10- OsbZIP86- OsNCED3 (Gao et al. 2022). As in the regulation of ACS and ACO, several reports suggest that MAPK may play a pivotal role in the post-transcriptional regulation of the key enzymes for ABA biosynthesis. In rice, for example, Zhang et al. (Zhang et al. 2022) demonstrated that OsMPK1, a MAPK, could phosphorylate OsABA2^S197^ in vitro, whereby enhancing the stability of OsABA2 protein. Additionally, it was shown that OsbHLH110, a basic helix-loop-helix transcription factor, could directly bind to the G-box element in the *OsABA2* promoter to enhance its expression. These observations suggest that OsABA2 can be regulated both transcriptionally and post-transcriptionally (Zhang et al. 2024a). Although these findings have been obtained in *Arabidopsis* and crop plants, they have provided valuable information for elucidating the mechanism of HSP of ABA.

## Cell biological mechanism of HSP

### Fertilization triggered production of negative signals

In terms of cell biological events, the whole process of fruit development and ripening can be divided into three major stages: the early developmental stage characterized by cell division, differentiation and slow growth/expansion; the mid-developmental stage characterized by the initiation of cell wall degradation and fast cell expansion; and the late developmental stage characterized by cell senescence and breakdown. As cell division, differentiation and growth are determined by a set of growth-promoting hormones, e.g. auxin (IAA), gibberellin (GA) and cytokinin (CTK), which are believed to be negative signals of fruit ripening, production of these hormones is required for early development immediately after fertilization.

Increasing evidences indicate that fertilization activates IAA biosynthesis. More than several decades ago, several studies reported the importance of IAA in the formation of somatic embryos (Steward et al. 1958; Reinert 1959; Michalczuk et al. 1992; Ribnicky et al. 1996). Later, a study by Ribnicky et al., reported a dramatic increase in the concentration of free IAA from a basal level of ca. 25 ng/g FW in unfertilized ovules to ca. 2,000 ng/g FW in the late globular and early heart stages after fertilization in carrots (Ribnicky et al. 2002). In rice, it was also found that the content of IAA greatly increased in the ovary following pollination/fertilization, subsequently IAA will transport from the ovary to the rachilla–pedicel after pollination (Uchiumi and Okamoto 2010). In recent years, a number of studies further explored the mechanism for fertilization-triggered IAA increase (Guo et al. 2022; Robert et al. 2018; Li et al. 2022a; Liu et al. 2023a). In *Arabidopsis*, local auxin sources was found to orient the apical-basal axis in embryos. (Robert et al. 2013; Liu et al. 2023a).

The maternal plant provides IAA to the early embryo from the integuments of the ovule, and IAA responses increases in ovules upon fertilization, due to upregulated IAA biosynthesis in the integument (Robert et al. 2018). Zhongchi Liu’s group conducted further researches on the molecular mechanism for fertilization-triggered IAA accumulation in strawberry, and demonstrated a type I MADS box gene *AGL62* is required for the activation of auxin synthesis in the endosperm and several *FveATHB* downstream of *FveAGL62* act to repress auxin biosynthesis (Guo et al. 2022). There is an interplay between IAA and GA as well as other negative signals (Serrani et al. 2007; Li et al. 2020; He and Yamamuro 2022). Overall, current studies have shown that IAA undoubtedly acts to be the earliest signal from fruit set to ripening.

### Seed cell dehydration-induced production of positive signals

It is well known that environmental stress-induced cellular water dehydration might have a strong effect on hormonal biosynthesis and catabolism, especially the induction of ABA and ET biosynthesis (Aharoni 1978; Apelbaum and Yang 1981; Wright 1981; Seo and Koshiba 2002; Verslues and Zhu 2005; Larrainzar et al. 2014; Komatsu et al. 2020). Notably, besides environmental stress, cell water dehydration may happen during some developmental processes with seed development serving as an example. Seed development from embryo formation to maturation is accompanied with a dramatic reduction of water content, decreasing from over 90% to below 10% (Lee et al. 2018; Vaz et al. 2018; Fonseca De Oliveira and Amaral Da Silva 2024). Consequently, both water potential and osmotic potential must significantly decrease during seed development (Welbaum and Bradford 1988; Welbaum and Bradford 1990). In *Pinus taeda*, for example, the seed osmotic potential was assessed to be decreased from −0.85 to −2.0 MPa from the stage of early seed development to maturation. Such a large decrease in the osmotic potential may surpass that caused by common environmental stresses, implying that seed development should be tightly linked with HSP in seeds. Consistently, ABA accumulation is well known to be accompanied with seed maturation (Sano and Marion-Poll 2021; Ali et al. 2022), which is necessary to arrest seeds’ precocious germination before harvest. In addition to the impact on ABA and ET biosynthesis, the cell dehydration during seed development may also have a strong effect on the modulation of HSP for other hormones, warranting compressive investigation in the future. Fruit functions primarily as an organ for seed dispersal; thus, its development and ripening must align with that of the seed. This indicates a necessity for signaling communication between the seed and the fruit. Investigating the triggering mechanism of HSP in seeds is crucial for comprehending the signaling processes involved in fruit development and ripening.

### Cell wall degradation and changes of membrane tension as triggers of HSP

Cell wall degradation is one of the most important events in fruit development and ripening (Jia et al. 2023). It starts with a breakdown of the middle lamina, and then, an overwhelming degradation of the whole wall occurs. Cell wall degradation enables cells to expand rapidly, owing to a release of the wall constraint, and this would result in a substantial change of the membrane tension (Zhang et al. 2020). Cell membrane is known to contain tension-sensitive Ca^2+^-channels (Moulia et al. 2015; Basu and Haswell 2017; Colin and Hamant 2021; Tyagi et al. 2023; Zhang et al. 2023d; Yu et al. 2024). It is reasonable to propose that the change of membrane tension would definitely trigger Ca^2+^ signaling. However, whether Ca^2+^ signaling may be linked with HSP is unknown.

Aside from the tension-triggered Ca^2+^ signaling, there are other mechanisms that may be implicated in HSP. A likely mechanism is the ‘cell wall integrity (CWI)’-associated signaling, in which one subfamily of the Receptor-Like Protein Kinase (RLK), FERONIA, has been thought to be capable of functioning as CWI sensors (Höfte 2015; Wolf 2017; Feng et al. 2018; Westermann et al. 2019; Zhou et al. 2024). There is evidence that FERONIA is implicated in fruit development and ripening (Jia et al. 2017a, 2017b, 2023). Importantly, two FERONIA-Like Receptor Kinases were identified to be implicated in the regulation of tomato fruit ripening via modulation of ET production (Jia et al. 2017b). Further, a study by Ji et al. demonstrated that SIFERL, a member of FERONIAs, could directly interact with ACS (S-Adenosylmethionine Synthetase, a key enzyme in ET biosynthesis) thereby regulating ET biosynthesis and tomato fruit ripening. It remains to demonstrate whether FERONIA activity can respond to the changes of CWI or membrane tension in fruit development and ripening.

**Fig. 5:**
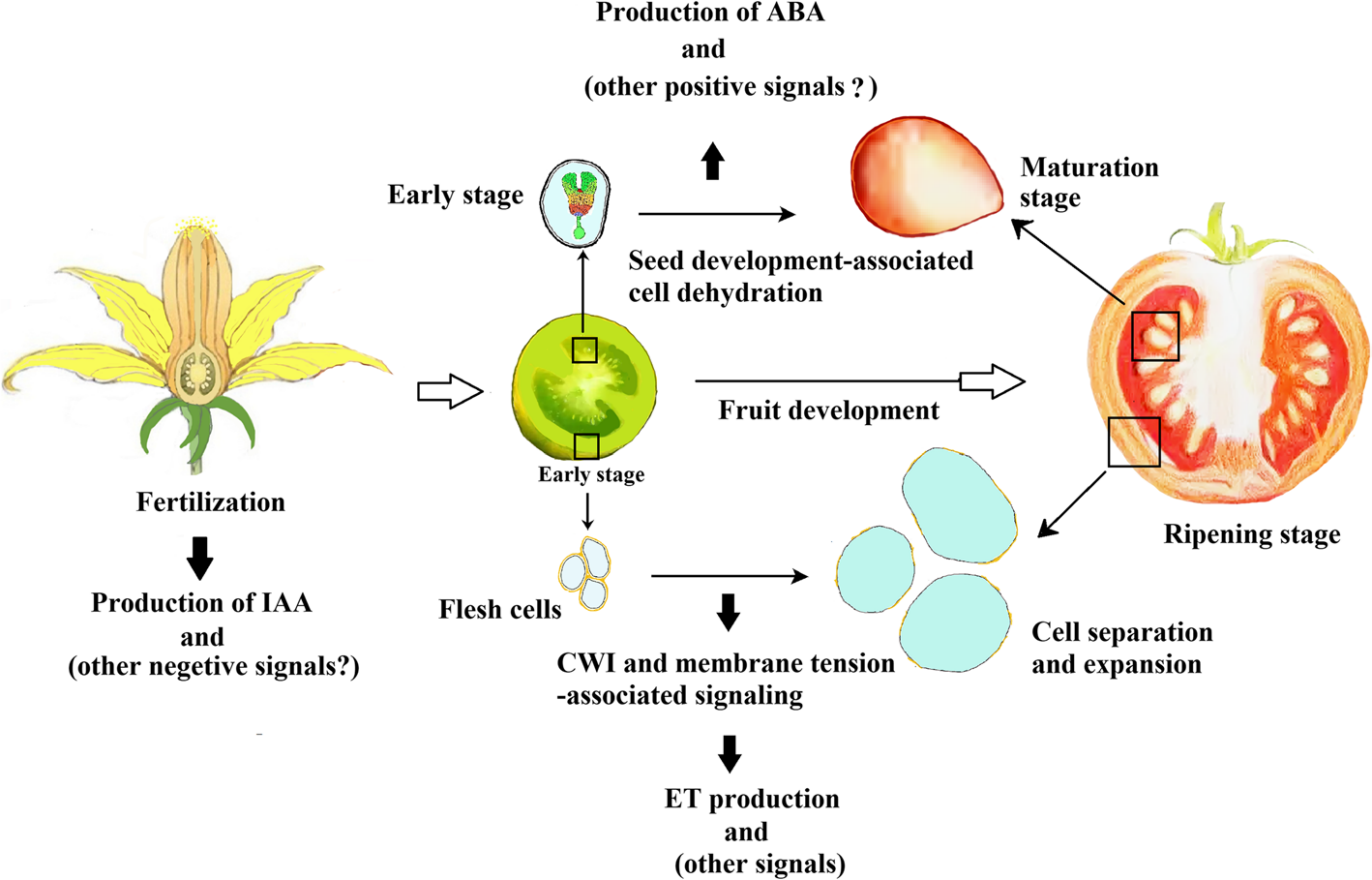
Diagram showing the cell biological mechanism of hormonal signal production. Fertilization triggers IAA production in the embryo. Development-associate cell dehydration triggers ABA production in the seed. Changes in CWI and membrane tension likely function as mechanisms to trigger ET production. The questioned words in brackets denote the signals potentially produced. Boxed are amplified areas. CWI, cell wall integrity

In summary (Fig. 5), emerging evidence indicates a strong association between HSP and various cellular biological events. While fertilization triggers the production of negative hormonal signals, among which IAA is the earlies signal. Additionally, the decreased water potential resulting from seed cell dehydration triggers the production of ABA signal as well as some other positive signals. In addition to fertilization and seed cell dehydration, fruit development-associated changes of CWI and membrane tension are could be the mechanisms for modulating HSP. On one hand, hormonal signals regulate various biological events (e.g. physiological and biochemical metabolisms and cell structure patterns), on the other hand, HSP arises from alterations in these biological events. So, there exists a relationship of chicken-and-egg between HSP and biological events.

## Conclusion and perspective

As positive signals, ET and ABA respectively promote CL and NC fruit ripening, whereas as a negative signal, IAA acts to promote fruit growth and antagonize the action of ET and ABA in the promotion of FF ripening. Functioning of these hormones is predicated by a sudden change in the hormonal levels throughout fruit development and ripening, which is essentially the process of HSP. In terms of its physiological and biochemical bases, HSP is determined by the functional proteins (e.g. enzymes and transporters) controlling hormone metabolism and transport. ACS and ACO function as the key enzymes for ET biosynthesis, whereas NCED, ABA2 and AAO3 function as the key enzymes for ABA biosynthesis. The IAA transport from seeds to flesh cells is critical to the orchestrated growth and maturation between seeds and fruit. Behaviors of the key enzymes (i.e., activity, stability and localization) are modulated by diverse transcription factors, which are gain regulated by a complex upstream signaling network, with MAPKs and CDPKs playing crucial roles in mediation of HSP. To further elucidate the mechanism of HSP, several perspectives are presented below to aid understanding of the prospective research.

### Hormonal communication between seeds and fruit maturation

In nature, fruit production is essentially for seeds’ dispersal. Hence, the process of fruit growth and ripening relies on precise coordination with that of seeds. The hormonal communication is expected to have a crucial function in the synchronized control of maturity between seeds and flesh. However, the specific mechanism the hormonal communication operates via is yet mostly unidentified. This should be clarified with the greatest emphasis in future research.

### Mechanism for the interplay between the production of ABA and IAA

ABA and IAA are respectively the core components of the positive and negative signals. On one hand, IAA was reported to activate ABA catabolism, consequently leading to the suppression of ABA accumulation (Liao et al. 2018), but on the other hand, there is evidence that ABA may act to suppress IAA accumulation, implying that the declined level of IAA was likely resulted from the ABA accumulation (Li et al. 2022b). The question lies in how the decrease in the level of flesh IAA is triggered at the onset of fruit ripening, and whether the decreased level of IAA may act to initiate the ABA accumulation during fruit ripening. In addition, as seeds’ maturation is always accompanied by a dramatic ABA accumulation, it is unclear whether the ABA accumulation in seeds may act to trigger the decrease in the level of IAA in seeds and flesh cells. Furthermore, the coordination between the ABA accumulation in seeds and the flesh remains unclear. These questions require to be clarified in the future.

### The mechanism for interplay between the production of ET and IAA

IAA has been suggested to induce ET production in diverse biological processes (Andreae et al. 1968; Burg and Burg 1968; Chadwick and Burg 1970; Sargent et al. 1974; Peck and Kende 1995). There is a report that IAA induced ET production might be involved in fruit ripening. For example, a study by Tatsuki et al. (Tatsuki et al. 2013) reported that IAA may act to trigger system II production in peach, but this proposal is based on the observation that an increase in the IAA level just coincided with system II ET production. There has been lack of essential evidences supporting the role of IAA in the initiation of ET production. More studies are required to confirm whether IAA accumulation definitely occurs at the onset of seeds’ maturation regardless of plant cultivars or varieties.

### The signaling cascades upstream of transcription factors

In terms of its physiological and biochemical bases, HSP is determined by the functional proteins controlling hormonal metabolism or transport. A significant number of transcription factors have been recognized as playing a role in metabolic regulation; however, only a limited number of signaling proteins upstream of these transcription factors have been identified (Tables 2 and 3). In addition, the signal amplification is a critical part of HSP for ET and ABA. To profoundly understand the mechanism of HSP for ET and ABA, it is essential to thoroughly decipher the signaling cascades from ET and ABA perception to the behavior regulation of the key enzymes in the ET and ABA biosynthesis pathway.

### The primary signals triggering HSP

The primary signal denotes the initial factor that activates HSP. Given that fertilization can be regarded as the earliest event of fruit development and ripening, whether the fertilization-induced IAA production acts to be primary signal needs to be clarified. Since it is the decrease rather than the increase in the IAA level that is coupled with fruit ripening, the primary signaling should be linked with the event that acts to trigger the decline of the IAA level. The key lies in whether the decreased level of IAA acts to trigger ABA and ET accumulation at the onset of fruit ripening. Fruit development-associated changes in osmotic potential or cell wall metabolism have been suggested to be a source of primary signals (Jia et al. 2020, 2023), accordingly, it should be paid more attentions in future studies on HSP. Notably, environmental factors significantly influence fruit ripening and can be considered primary signals. The mechanisms for the triggering of HSP by environmental factors is worthy of studies in the future.

### The mechanism for fertilization triggered production of negative signals

Fertilization is the earliest event in fruit development and ripening; and accordingly, fertilization-triggered HSP is undoubtedly the primary signal implicated in the regulation of fruit ripening. Although it is clear that IAA production is triggered by fertilization, whether fertilization may trigger the production of other negative signals, such as GA and CYT, remains elusive. It is essential to clarify whether fertilization may directly trigger the production of GA and CYT, or whether the initial production of IAA acts to trigger the production of GA and CYT. Finally, it is of great interest and importance to further decipher the signaling mechanism underlying fertilization-triggered production of IAA as well as other negative signals.

### The mechanism for seed development-associated production of positive signals

Environmental stresses, particularly cell dehydration, can significantly influence hormonal biosynthesis and catabolism. Notably, seed development is essentially a process of cell dehydration, and accordingly, it is reasonable to propose that seed development must be accompanied with HSP. This is particularly true for ABA signal production, because ABA accumulation is required to inhibit pre-harvest sprouting. Besides ABA accumulation, further study is essential to clarify whether seed dehydration may trigger the production of other positive signals, such as ET, JA and etc. Also, it is of great interest and importance to decipher the signaling mechanism for the seed dehydration-induced positive signals.

### The mechanism for CWI and membrane tension-associated signaling in relation to HSP

Fruit development and ripening are accompanied by cell degradation, and this would inevitably interfere with cell wall integrity (CWI) and membrane attention. Plants have evolved mechanisms for sensing CWI and membrane attention. Receptor-like protein kinase (RLK) and tension-sensitive Ca^2+^ channels have been demonstrated to be crucial sensors of CWI and membrane tension (Zhou et al. 2024; Westermann et al. 2019; Feng et al. 2018; Wolf 2017; Höfte 2015). Studies are essential to further demonstrate whether RLK, particularly FERONIA and Ca^2+^ signaling may be involved in the production of the positive signals.

## Data Availability

Not Applicable to this article as no datasets were generated or analysed during the current study.

## Abbreviations

1-MCP: 1-Methylcyclopropene
AAO: Abscisic acid aldehyde oxidase
ABA: Abscisic acid
ABA2: Abscisic acid biosynthesis protein 2
ABA-GE: β-D-glucopyranosyl abscisate
ABAUGT: AOG/ABA-uridine dihosphate glucosyltransferase
ABC: ATP-binding cassette
ABCG: ATP-binding cassette (ABC) G-family proteins
ACC: 1-Aminocyclopropane-1-carboxylic acid
ACO: 1-Aminocyclopropane-1-carboxylate oxidases
ACS: 1-Aminocyclopropane-1-carboxylate synthases
AO: Aldehyde oxidase
BG: β-Glucosidase
BR: Brassinosteroid
CDPK: Calcium dependent protein kinase
CL: Climacteric
CTK: Cytokinin
CWI: Cell wall integrity
CYP707A: Abscisic acid 8’-hydroxylase
CYPAs: Cytochrome P450
CYT: Cytokinin
DPA: Dihydrophaseic acid
ET: Ethylene
FF: Fleshy Fruit
FMO: Flavin-containing monooxygenases
FZY: Flavin monooxygenase
GA: Gibberellin
HAT1: Histone acetyltransferase 1
HSP: Hormonal signal production
IAA: Indole-3-acetic acid
IAM: Indole-3-acetamide
IAOX: Indole-3-acetaldoxime
IPyA: Indole-3-pyruvate acid
JA: Jasmonic acid
MAPK: Mitogen activated protein kinase
MEP: Methylerythritol 4-phosphate
MKK: Mitogen-activated protein kinase kinase
NAA: 1-Naphthaleneacetic acid
NC: Non-climacteric
NCED: 9-Cis-epoxycarotenoid dioxygenase
PA: Phaseic acid
PP2C: Type 2C protein phosphatase
RLK: Receptor-like protein kinase
SAM: S-adenosylmethionine
SDR: Short-chain alcohol dehydrogenase/reductase
SnRK2s: Sucrose non-fermenting 1-related protein kinase SRK2E
TAA1: Tryptophan-pyruvate aminotransferase 1
TAM: Tryptamine
YUCCA: Flavin-binding monooxygenase family protein
ZEP: Zeaxanthin epoxidase
